# A Cell-Permeable Fluorescent Polymeric Thermometer for Intracellular Temperature Mapping in Mammalian Cell Lines

**DOI:** 10.1371/journal.pone.0117677

**Published:** 2015-02-18

**Authors:** Teruyuki Hayashi, Nanaho Fukuda, Seiichi Uchiyama, Noriko Inada

**Affiliations:** 1 The Graduate School of Biological Sciences, Nara Institute of Science and Technology, Ikoma-shi, Nara, Japan; 2 Graduate School of Pharmaceutical Sciences, The University of Tokyo, Bunkyo-ku, Tokyo, Japan; University of Wurzburg, GERMANY

## Abstract

Changes in intracellular temperatures reflect the activity of the cell. Thus, the tool to measure intracellular temperatures could provide valuable information about cellular status. We previously reported a method to analyze the intracellular temperature distribution using a fluorescent polymeric thermometer (FPT) in combination with fluorescence lifetime imaging microscopy (FLIM). Intracellular delivery of the FPT used in the previous study required microinjection. We now report a novel FPT that is cell permeable and highly photostable, and we describe the application of this FPT to the imaging of intracellular temperature distributions in various types of mammalian cell lines. This cell-permeable FPT displayed a temperature resolution of 0.05°C to 0.54°C within the range from 28°C to 38°C in HeLa cell extracts. Using our optimized protocol, this cell-permeable FPT spontaneously diffused into HeLa cells within 10 min of incubation and exhibited minimal toxicity over several hours of observation. FLIM analysis confirmed a temperature difference between the nucleus and the cytoplasm and heat production near the mitochondria, which were also detected previously using the microinjected FPT. We also showed that this cell-permeable FPT protocol can be applied to other mammalian cell lines, COS7 and NIH/3T3 cells. Thus, this cell-permeable FPT represents a promising tool to study cellular states and functions with respect to temperature.

## Introduction

Temperature is a fundamental physical parameter related to many cellular functions, including gene expression, protein stabilization, enzyme-ligand interactions and enzyme activity [1]. Intracellular temperatures fluctuate depending on the chemical reactions occurring inside cells, which are accompanied by either heat release (exothermic) or heat absorption (endothermic), as well as on changes in the ambient temperature. An accurate method for directly measuring intracellular temperatures could provide information regarding the status of a cell; thus, the development of novel cellular thermometers has been of great interest [2–5].

To provide a basis to study the relationship between temperature and cellular functions, we previously developed a fluorescent thermometer capable of measuring the intracellular temperature distribution with high spatial (~200 nm) and temperature resolution (0.18°C-0.58°C in the range of 29–39°C) [6]. This method utilized a novel fluorescent polymeric thermometer (FPT) in combination with fluorescence lifetime imaging microscopy (FLIM). The FPT consists of a thermosensitive poly(*N*-*n*-propylacrylamide) (polyNNPAM) unit, an ionic unit (potassium 3-sulfopropyl acrylate, SPA) and a water-sensitive fluorescent unit (*N*-{2-[(7-*N*,*N*-dimethylaminosulfonyl)-2,1,3-benzoxadiazol-4-yl](methyl)amino}ethyl-*N*-methylacrylamide, DBD-AA). At lower temperatures, the thermosensitive unit adopts an extended conformation due to the hydration of amide linkages; this hydration weakens at higher temperatures, thus causing the thermosensitive unit to shrink. The DBD-AA fluorescence is quenched in the presence of neighboring water molecules. Upon the shrinkage of the thermosensitive unit in response to increased temperature, these water molecules are expelled, resulting in increased DBD-AA fluorescence [6]. The SPA unit enhances the hydrophilicity of the FPT, thus preventing the interpolymeric aggregation of the FPT within cells [7]. This FPT displays both increased fluorescence intensity and longer fluorescence lifetime with increasing temperature [6]. The fluorescence lifetime is a parameter that is independent of experimental conditions such as the concentration of the FPT or the excitation power [8] and is thus suitable for measuring temperature-dependent changes in FPT fluorescence inside the cell, where precisely controlling the concentration of an introduced fluorescent probe is difficult. Using this fluorescent thermometer, we provided the first evidence of an intracellular temperature difference: the temperature in the nucleus was approximately 1°C higher compared with the cytoplasm in mammalian cell lines. The temperature difference between the nucleus and the cytoplasm changed depending on the cell cycle status, suggesting a correlation between cellular function and temperature. In addition, the areas surrounding the centrosome and the mitochondria exhibited higher temperatures than other areas of the cytoplasm [6].

However, this previous method required microinjection to deliver the FPT into living cells. For experimental convenience, cell-permeable FPTs are desirable. Recently, we reported the development of the novel fluorescent thermometer NN-AP2.5, which is a polymeric thermometer consisting of an NNPAM unit, a cationic 3-(acrylamidopropyl)trimethylammonium (APTMA) unit, and a DBD-AA unit [9]. This probe was developed for measuring intracellular temperatures in yeast cells, whose cell wall hinders microinjection. NN-AP2.5 spontaneously and rapidly diffused into living yeast cells (within less than 20 min of incubation) and measured cellular temperature with a 0.09–0.78°C temperature resolution in the range from 15°C to 35°C. Furthermore, NN-AP2.5 displayed spontaneous cellular entry into mammalian MOLT-4 and HEK293T cells [9]. Although NN-AP2.5 was shown to function inside these cells, i.e., the fluorescence intensity increased and the fluorescence lifetime of NN-AP2.5 elongated upon an increase in the temperature, the intracellular temperature distribution has yet to be examined.

In this study, we report a novel FPT that displays both cell permeability and high photostability. The fluorescent DBThD-AA unit is an environmentally sensitive intramolecular charge transfer-type fluorophore, in which the oxygen atom of the 2,1,3-benzoxadiazole moiety in DBD-AA was replaced with a sulfur atom. This change has been shown to increase the photostability of fluorescent thermometers in aqueous solution by up to 10-fold compared with probes containing DBD-AA [10]. We optimized the protocol for introducing this cell-permeable FPT containing a DBThD-AA unit into HeLa cells and showed that this probe spontaneously diffused into living HeLa cells within 10 min of incubation. FLIM analysis of the cell-permeable FPT in HeLa cells revealed an intracellular temperature difference between the nucleus and the cytoplasm, as well as heat production near the mitochondria, in accordance with our previous report using the microinjected FPT [6]. We also confirmed that this cell-permeable FPT protocol is effective for other mammalian cell lines, although the optimal concentration of the FPT required for delivery differed between the cell lines.

## Materials and Methods

### Preparation of FPTs

FPTs were synthesized via random polymerization. For preparation of FPTs and the control copolymer, an *N*-alkylacrylamide-type monomer (NNPAM or NIPMAM) and the ionic monomer APTMA or SPA (total 2.5 mmol), the fluorescent monomer (DBD-AA or DBThD-AA) (25 μmol), and AIBN (25 μmol) were dissolved in *N*,*N*-dimethylformamide (5 mL), and the solution was bubbled with dry Ar for 30 min to remove dissolved oxygen. The solution was heated to 60°C for at least 8 h and then cooled to room temperature. The reaction mixture was poured into diethyl ether (100 mL). The resulting copolymers were purified via dialysis and characterized via 1H NMR, UV-Vis absorption, and gel-permeation chromatography (GPC) analyses. The reaction yields, the actual compositions, and the molecular weights of the polymers used in this study are shown in Table A in S1 File. The size and zeta potential of polymers were measured using a Zetasizer Nano ZS (Malvern, Worcestershire, UK) and are shown in Tables B and C, respectively, in S1 File.

### Measurement of temperature-dependent changes in the fluorescence intensities and the fluorescence lifetimes of cell-permeable FPTs in cell extracts

HeLa cell extracts were prepared as previously reported [6]. To examine the temperature-dependent changes in the fluorescence intensities of FPTs, 0.001 w/v% of FPTs in HeLa cell extracts was excited at 450 nm, and the fluorescence at 570 nm was measured using an FP-6500 spectrofluorometer (JASCO, Tokyo, Japan) equipped with an R-7209 optional photomultiplier tube (operational range: 200–850 nm, Hamamatsu, Shizuoka, Japan,) as described previously [7]. The temperature-dependent change in the fluorescence lifetime of 0.02 w/v% FPTs in HeLa cell extracts was analyzed using a FluoroCube 3000U spectrofluorometer (HORIBA Jobin Yvon, Kyoto, Japan) with an ETC-273T temperature controller (JASCO). The sample was excited with a pulsed diode laser (NanoLED-405L, Horiba, 405 nm) at a repetition rate of 1 MHz, and the emission longer than 500 nm was collected [6]. The obtained fluorescence decay curve was analyzed by fitting the curve by a double exponential function using the following equation:

$$\tau_{\text{f}}=\frac{\left({\text{A}}_{1}\tau_{1}+{\text{A}}_{2}\tau_{2}\right)}{{\text{A}}_{1}+{\text{A}}_{2}}$$

The calibration curve for the temperature imaging of HeLa cells with the FPT was obtained by approximating the relationship between the averaged fluorescence lifetime of the FPT in HeLa cell extract (in triplicate) and the temperature to the sixth-degree polynomial (correlation coefficient r = 0.996):

$$\tau_{\text{f}}(T)=-1.323\times 10^{-6}T^{6}+2.63\times 10^{-5}T^{5}-2.166\times 10^{2}T^{4}+9.451\times 10^{-1}T^{3}-2.303\times 10T^{2}+2.974\times 10^{2}T-1.586\times 10^{3}$$

where *T* and τ_f_ (*T*) represent the temperature (°C) and the fluorescence lifetime (ns) at *T*°C, respectively. The temperature resolution (δT) of the FPT was evaluated by the following equation:

$$\text{δ}T=\;(\frac{\partial \text{T}}{\partial \tau_{\text{f}}})\text{δ}\tau_{\text{f}}$$

where ∂*T*/∂τ_f_ and δτ_f_ represent the inverse of the slope in the fluorescence lifetime-temperature diagram and the standard deviation of the averaged fluorescent lifetime, respectively.

### Introduction of the cell-permeable FPTs into mammalian cell lines

HeLa cells were cultured on a 35-mm glass dish (glass 27 ϕ, Iwaki, Japan) in high-glucose Dulbecco’s modified Eagle’s medium (DMEM; Nacalai Tesque, Kyoto, Japan) supplemented with FBS (Hyclone SH30910.03, Lot# AVB64834, Thermo Scientific, Japan) at 37°C with 5% CO_2_. For loading of cell permeable FPT into cells, DMEM medium was removed from a dish containing cells at 30 to 50% confluency, and cells were rinsed with 1 mL of 1×PBS (10×PBS contains 28.9 g of Na_2_HPO_4_–12H_2_O, 2.0 g of KH_2_PO_4_, 80.0 g of NaCl, and 2.0 g of KCl in 1 L solution). Then PBS was replaced with 1 mL of cell-permeable FPT at appropriate concentration in 5 w/v% glucose in water (for 0.01 w/v % of FPT, 2 µL of 5 w/v% FPT stock solution in water was diluted in 998 µL of 5 w/v% glucose in water. 5 w/v% stock solution in water was prepared and incubated at 4°C at least overnight before use to obtain full extension of the polymer). The dish was incubated at either 4°C or 25°C for 5 to 20 min without CO_2_ supply. After incubation, FPT solution was removed, and cells were rinsed with 1 mL of 1×PBS three times. 2 mL of phenol red-free DMEM medium (Gibco No. 21063, Life Technologies Japan, Tokyo, Japan) was added to the dish before imaging.

### Fluorescence imaging of cells

Confocal fluorescence imaging was performed using a laser scanning confocal microscope (Leica TCS-SP5, Leica, Germany) equipped with an HCX PL APO Ibd.BL 63×1.4 N.A. oil objective (Leica). Cells loaded with FPT was excited by 458 Argon laser, then fluorescence images were acquired through bandpass 500–700 nm in a 1024×1024 pixel format, with zoom factors ranging from 1 to 10 and scanning speed 400 Hz. The contrast and brightness of fluorescence images were enhanced using Adobe Photoshop for presentation. The incorporation efficiencies (%) of the FPTs were determined by counting the percentage of fluorescent cells per total number of cells in the fields (70 to 280 cells per field). For the co-visualization of temperature and mitochondria, cells were stained with 50 nM MitoTracker Deep Red FM (Life Technologies Japan) in phenol red-free DMEM medium for 5 min at room temperature, then treated with FPT. 458 Argon laser was used for excitation of FPT, and 633 HeNe laser was used for excitation of MitoTracker Deep Red FM. The fluorescence of FPT was collected through bandpass 500–600 nm, and the fluorescence of MitoTracker Deep Red FM was collected through bandpass 645–730 nm.

### Evaluation of the cytotoxicity of the cell-permeable FPT

To examine the cell proliferation rate, HeLa cells at 30% confluency were treated with 5 w/v% glucose solution in water containing 0.01 w/v% FPT at 25°C for 10 min were incubated in culture medium at 37°C in 5% CO_2_. After 1, 6 or 24 h of incubation, the number of cells was counted under a microscope (Nikon ECLIPSE TS100, Nikon, Japan) equipped with 10× 0.25 N.A. objective (Nikon). Cell viability was assessed via propidium iodide (PI, Sigma-Aldrich Japan, Tokyo, Japan) staining. After treatment with a cell-permeable FPT, the cells were incubated for 1, 3 or 6 h at 37°C in 5% CO_2_, followed by treatment with PBS containing 500 nM PI for 10 min at room temperature. The cells were rinsed twice with PBS and then observed under a TCS-SP5 confocal microscope (Leica). The cells were excited at 488 nm, and the fluorescence of PI at 690–720 nm was recorded. The number of cells containing unstained nuclei was counted per 100 cells.

### Fluorescence lifetime imaging microscopy (FLIM)

For intracellular temperature imaging, the temperature of the microscope stage was regulated using an INUB-F1 controller (Tokai Hit, Shizuoka, Japan), and the temperature of the medium was monitored using a thermocouple probe (TSU-0125 thermometer equipped with a TSU-7225 probe, Tokai Hit). A TCS-SP5 confocal laser-scanning microscope (Leica) equipped with a 405 laser (PDL 800-B, PicoQuant, Berlin, Germany) and TCSPC module SPC-830 (Becker & Hickl, Berlin, Germany) was used for FLIM analysis. The pulse repetition rate of 405 laser was set at 20 MHz. The fluorescence was captured through an HCX PL APO Ibd.BL 63× 1.4 N.A. oil objective (Leica) with zoom factor ranging from 1 to 10 in 64×64 pixel format at 400 Hz scanning speed (scanning duration was set for 60 seconds) through bandpass 500–700 nm for cells loaded with FPT only, and 500–550 nm for cells co-stained with MitoTracker Deep Red FM. The laser power, sensitivity of the detector, and pinhole size were controlled so that the photon count rate does not exceed 2% of pulse count rate (2×10^7^). The obtained fluorescence decay curve in each pixel was fitted with a double exponential function using SPCImage software (Becker & Hickl) after the binning procedure (factor: from 2 to 6).

### Treatment of cells with an uncoupling reagent

10 µL of 2 mM carbonyl cyanide 3-chlorophenylhydrazone (CCCP, Sigma-Aldrich) in DMSO was added to 2 mL of phenol red-free DMEM medium in a glass base dish containing HeLa cells loaded with FPT. The temperature of the culture medium was maintained at 30°C during analysis. For a control, 10 µL of DMSO was added.

## Results

### Characterization of the cell-permeable FPTs

Recently, we reported that the FPT containing an APTMA unit spontaneously incorporated into yeast cells as well as both non-adherent (MOLT-4, human acute lymphoblastic leukemia cell line) and adherent (HEK293T, human embryonic kidney cell line that contains SV40 large T-antigen) mammalian cell lines. The incorporation efficiency increased as the ratio of the APTMA unit in the FPT increased (e.g., NN-AP2.5, in which the ratio was NNPAM:APTMA = 97.5:2.5, displayed a lower incorporation efficiency than NN-AP25, in which the ratio was NNPAM:APTMA = 75:25) [9]. In contrast, the temperature sensitivity decreased as the proportion of APTMA in the FPT increased [9].

**Fig 1:**
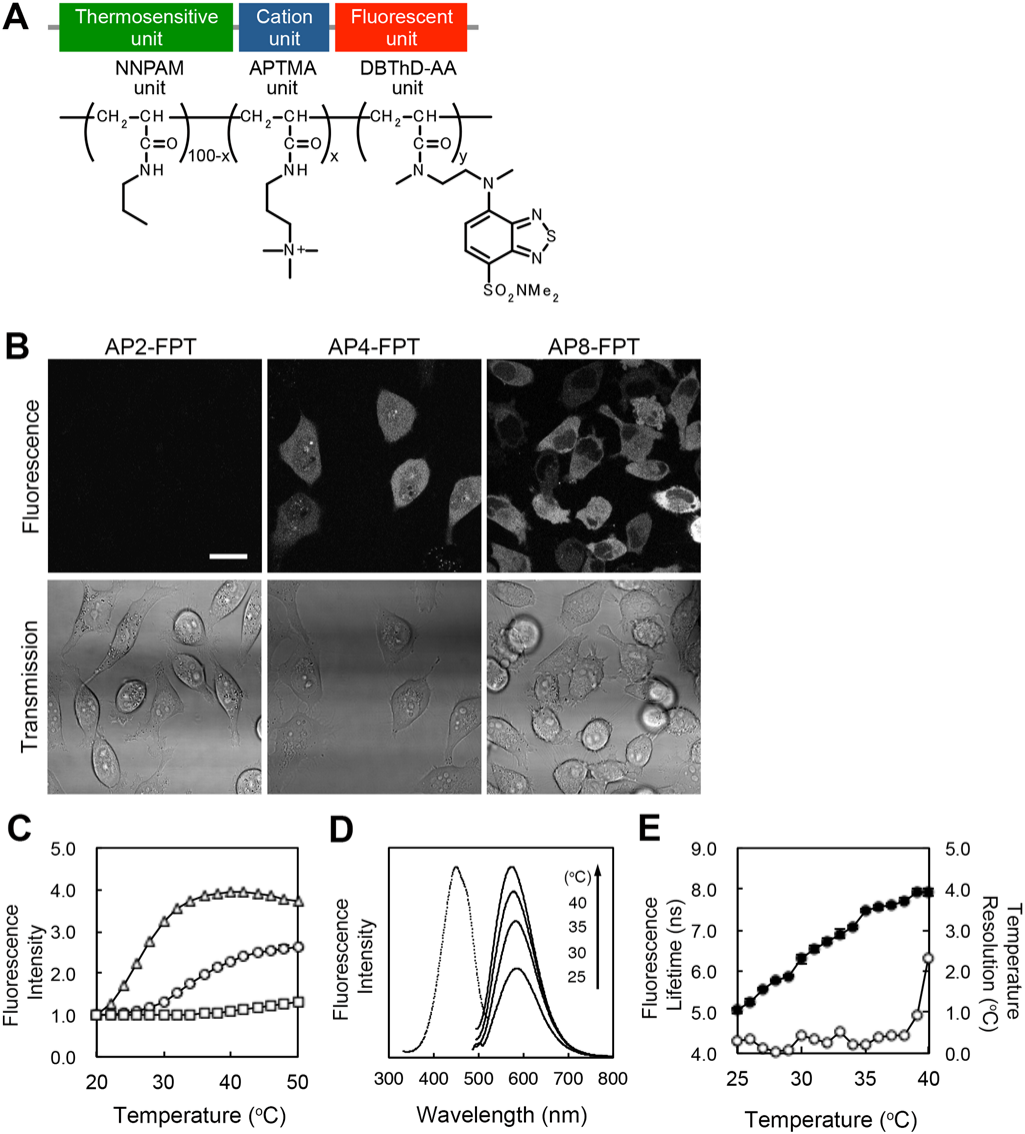
Cell-permeable and highly photostable FPTs for intracellular temperature mapping. A) Chemical structure of the cell-permeable fluorescent polymeric thermometer (FPT). The original name of each unit is described in the main text. Numbers at each unit indicate the proportion of each unit in the copolymer. B) Confocal fluorescence (top) and transmission microscopy images (bottom) of HeLa cells treated with the FPTs. Scale bar = 20 µm. C) Temperature-dependent changes in the fluorescence intensity of the cell-permeable AP2-FPT (triangle), AP4-FPT (circle) and AP8-FPT (square) in HeLa cell extracts. The vertical bars (behind the plots) indicate the s.d. based on triplicate measurements. The fluorescence intensities were normalized at 20°C. D) Fluorescence spectra of AP4-FPT in HeLa cell extract. An excitation spectrum (dotted, at 40°C) was obtained from emissions at 573 nm. Emission spectra (solid) were obtained with an excitation at 450 nm. E) Temperature-dependent changes in the fluorescence lifetime of the AP4-FPT (solid circle) and the temperature resolution (open circle) in HeLa cell extracts. The vertical bars (behind the plots) indicate the s.d. based on triplicate measurements.

To develop a protocol for intracellular temperature mapping using a cell-permeable FPT containing DBThD, we first optimized the proportion of the APTMA unit in the FPT. For optimization, both the temperature sensitivities and the incorporation efficiencies of FPTs containing different APTMA ratios (x = 2, 4 or 8 in APx, Fig. 1A) were analyzed (Figs. 1B and 1C). To analyze the incorporation of FPTs into HeLa cells, a widely used adherent mammalian cell line derived from human cervical cancer cells, cells were treated with 0.05 w/v% cell-permeable FPT in 5 w/v% glucose in water (the conditions used in [9]) for 10 min at 25°C and observed via confocal microscopy. The incorporation efficiency increased as the proportion of APTMA in the FPT increased; AP2-FPT displayed very little incorporation, whereas many cells incubated with AP4-FPT and most of the cells incubated with AP8-FPT were fluorescent (Fig. 1B). The temperature-dependent changes of the fluorescence intensity in HeLa cell extracts revealed that the temperature sensitivity decreased as the proportion of APTMA increased (Fig. 1C). The effects of changing the APTMA ratio on the incorporation efficiency and the temperature sensitivity were in agreement with our previous findings [9]. In addition, the temperature range that could be resolved differed between these FPTs; the change in the fluorescence intensity of AP2-FPT became saturated at temperatures above 30°C, whereas AP4-FPT responded to a temperature range between 25 and 40°C (Figs. 1C and 1D), which is the optimal temperature range for examining mammalian cell lines. AP8-FPT displayed almost no response to temperature (Fig. 1C).

Based on these results, we chose AP4-FPT for further characterization and application for intracellular temperature mapping, as this probe displayed sufficient temperature sensitivity and cellular incorporation efficiency, and references to the cell-permeable FPT or FPT hereafter correspond to AP4-FPT. The fluorescence lifetime of the FPT displayed temperature-dependent elongation (Fig. 1E and Fig. A part A in S1 File), and the temperature resolutions, calculated based on the fluorescence lifetime, were 0.05 to 0.54°C within the range from 28 to 38°C (Fig. 1E) when measured with a spectrofluorometer (see Materials and Methods). The temperature resolution measured with FLIM (see Materials and Methods in S1 File) was 0.30 to 1.29°C within the range from 25 to 35°C (Fig. A part A in S1 File). As evidenced by variation of the fluorescence lifetime in a field of view (Fig. B in S1 File), the temperatures of the sample in a glass bottom dish on the microscope stage was likely not evenly regulated, probably due to a fluctuation of the temperature of the microscope stage, which could also be affected by a fluctuation of the room temperature regulated by the air conditioner. As both spectrofluorometer and FLIM gave similar fluorescence lifetimes (Fig. A part B in S1 File), we used the calibration curve obtained by the spectrofluorometer for conversion of the fluorescence lifetime into the temperature.

The temperature sensitivity of FPT was not significantly affected by environmental pH (5.78–9.39), ionic strength (in the range of 0.25–0.35), and viscosity (0–40 mg/mL Ficoll) at values that include the ranges of physiological variations in the cytosol [6,11,12] (Fig. C in S1 File).

### Optimization of the protocol for introducing the cell-permeable FPT into HeLa cells

**Fig 2:**
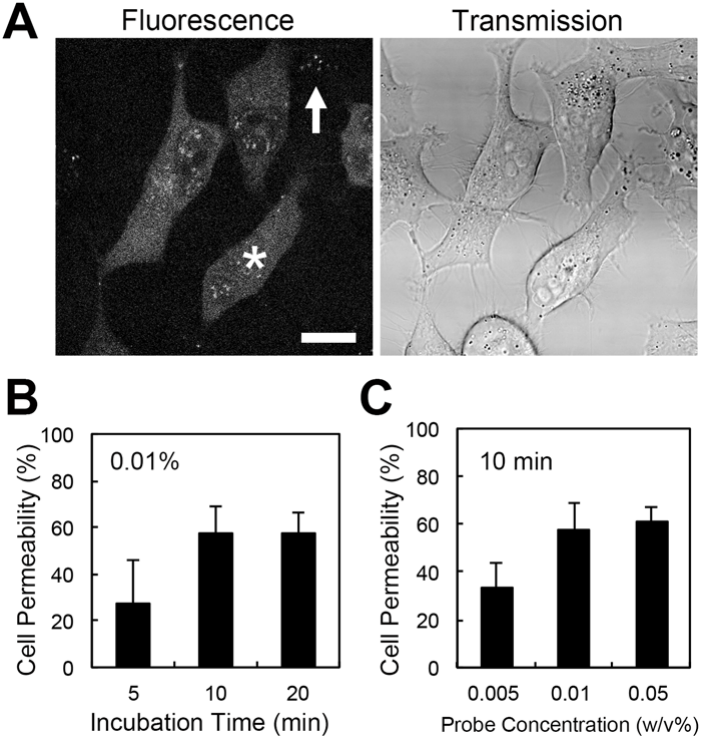
Optimization of the experimental conditions to introduce the cell-permeable FPT into HeLa cells. A) Confocal fluorescence and transmission microscopy images of living HeLa cells treated with the cell-permeable FPT. The FPT was distributed throughout the cytoplasm and the nucleus in most cells (a representative cell is indicated by an asterisk). Some cells displayed a punctate pattern of fluorescence (indicated by an arrow). Scale bar = 10 µm. B) The incorporation efficiency of the cell-permeable FPT was plotted against the duration after treatment with a solution containing 0.01 w/v% FPT in 5 w/v% glucose. C) The concentration-dependence of the incorporation efficiency of the FPT into cells. HeLa cells were treated with the indicated concentration of FPT in 5 w/v% glucose for 10 min. The vertical bars indicate the s.d. based on three independent experiments.

To optimize a protocol for delivering the cell-permeable FPT into living cells, we examined several incubation parameters: the temperature, the composition of the incubation solution, the FPT concentration, and the incubation duration. When the cells were analyzed under a microscope, two types of incorporation patterns were observed. In most cells, the fluorescence was distributed throughout the cell (indicated by an asterisk in Fig. 2A), whereas some cells displayed fluorescent puncta (indicated by an arrow in Fig. 2A). The percentage of cells containing fluorescent puncta out of all fluorescent cells was approximately 10%. Hereafter, only the cells containing distributed fluorescence were counted as cells in which the FPT was incorporated.

First, we analyzed the effect of changes in the ambient temperature during incubation to the incorporation efficiency. When cells were incubated with 0.01 w/v% FPT in 5 w/v% glucose solution at 25°C, the incorporation efficiency was approximately 60%; at 4°C, the incorporation efficiency was slightly reduced but remained higher than 40% (Table 1). In the following experiments, the incubation temperature was set at 25°C.

**Table 1:** Effects of the solution composition and the temperature on the incorporation efficiency of the cell-permeable FPT into HeLa cells.

Solution	Temperature (°C)	Cell permeability (%)^a^
5 w/v% glucose	25	57.7 ± 11.0
5 w/v% glucose	4	42.6 ± 0.77
DMEM	25	0
PBS	25	0

^a^Mean ± s.d., *n* = 3

The incorporation of FPT into HeLa cells was markedly affected by the composition of the incubation solution (Table 1). Ionic solutions, such as DMEM and PBS, completely inhibited the incorporation of FPT into HeLa cells, whereas efficient incorporation was achieved using a non-ionic 5 w/v% glucose in water. 5 w/v% glucose solution itself did not induce cell permeability, as an anionic FPT with a SPA ionic unit and a DBThD fluorescent unit (Fig. D part A in S1 File) was not incorporated into HeLa cells when incubated with cells in 5 w/v% glucose solution (Fig. D part B in S1 File). No noticeable damage to the cells was induced by incubation of cells in 5 w/v% glucose solution (Fig. D part B in S1 File).

Next, the incubation duration was optimized (Fig. 2B). When HeLa cells were incubated with 0.01 w/v% FPT in 5 w/v% glucose at 25°C, the cell-permeable FPT was spontaneously incorporated into 28% of the cells within only 5 min after treatment. The number of fluorescent cells increased when the incubation period was extended to 10 min; however, further extension of the incubation period to 20 min did not significantly increase the incorporation efficiency (Fig. 2B).

To examine the effect of the cell-permeable FPT concentration on cellular uptake, HeLa cells were incubated with various FPT concentrations (0.005, 0.01 and 0.05 w/v%) in 5 w/v% glucose for 10 min at 25°C. The incorporation efficiency increased when the FPT concentration was increased from 0.005 to 0.01 w/v% but did not increase further when the concentration was increased to 0.05 w/v% (Fig. 2C). Incubating with a higher FPT concentration (0.1 w/v%) induced cell death, as evidenced by plasma membrane rupture, indicating the cytotoxicity of this FPT at high concentrations.

Based on these results, we concluded that treatment with 0.01 w/v% cell-permeable FPT in 5 w/v% glucose for 10 min at 25°C is optimal for introducing this fluorescent thermometer to HeLa cells.

### Evaluating the cytotoxicity of the cell-permeable FPT in HeLa cells

**Fig 3:**
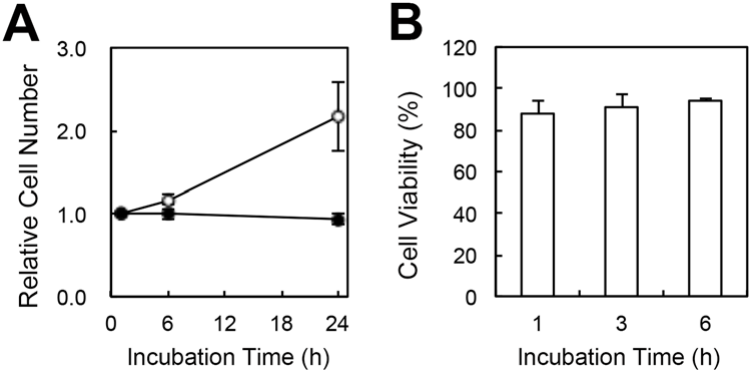
Effect of the cell-permeable FPT on cell proliferation and cell viability in HeLa cells. A) Cell proliferation was determined by direct cell counting at the indicated time intervals after treatment with (solid circle) or without (open circle) 0.01 w/v% FPT. The Y-axis indicates the cell number relative to the number at 1 h after incubation. B) Cell viability was determined by staining with propidium iodide (PI) as a marker of cell death at 1, 3 or 6 h after treatment with 0.01 w/v% FPT. The percentages of cells lacking PI-staining out of all counted cells are shown. The vertical bars indicate the s.d. based on three independent experiments.

As described above, the cell-permeable FPT was cytotoxic at high concentrations. To evaluate the cytotoxicity of this FPT, we examined cell proliferation and cell viability after treatment with 0.01 w/v% FPT for 10 min at 25°C. As shown in Fig. 3A, the number of mock-treated cells doubled in 24 h, whereas the FPT-treated cells did not exhibit any change in cell number (Fig. 3A). In addition to the lack of proliferation of the FPT-treated cells, the number of adherent cells slightly decreased. These results suggest that the introduction of the cell-permeable FPT inhibits cell proliferation and weakens cell adhesion.

Propidium iodide (PI) staining is generally used to assess damage to the plasma membrane and serves as a marker of cell death [13]. Fig. 3B shows the percentage of cells lacking PI staining. Most of the cells were not stained with PI, suggesting that those cells were viable, and this high cell viability did not change up to 6 h after treatment with the cell-permeable FPT. Thus, although the FPT inhibited cell proliferation, it exhibited low cytotoxicity even after 6 h of treatment.

### Imaging the intracellular temperature of living HeLa cells

**Fig 4:**
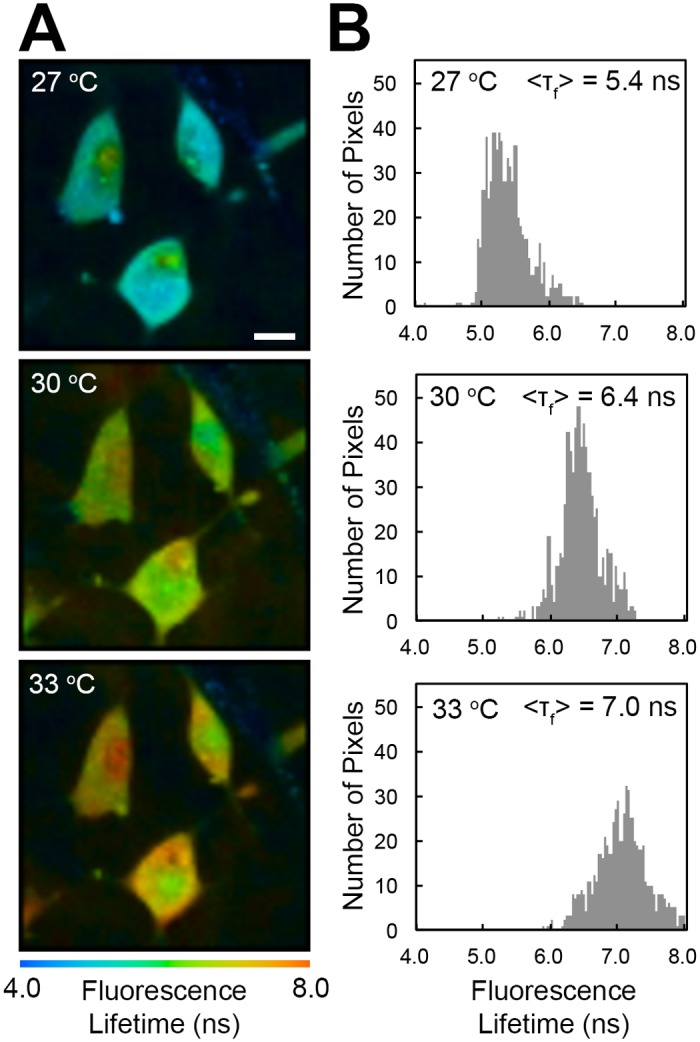
Temperature imaging of living HeLa cells via FLIM using the cell-permeable FPT. A, B) Fluorescence lifetime images of the FPT (A) and histograms of the fluorescence lifetime in cells (B). The temperature of the culture medium is indicated in each image. <τ_f_> indicates the average fluorescence lifetime in cells shown in A. Scale bars = 10 µm.

Next, we examined the functionality of the cell-permeable FPT in living HeLa cells via FLIM. The fluorescence lifetime of each pixel within the HeLa cells was prolonged as the temperature of culture medium was increased (Fig. 4). To confirm the temperature specificity of this FPT, we prepared a control copolymer consisting of an NIPMAM (*N*-isopropylmethacrylamide) unit instead of an NNPAM unit, along with the APTMA and DBThD units (Fig. E part A in S1 File). The only difference between the cell-permeable FPT and this control copolymer was the number of carbon atoms in the thermosensitive unit. This control copolymer did not display any temperature-dependent changes in either the fluorescence intensity or the fluorescence lifetime in HeLa cell extracts (Fig. E parts B and C in S1 File) but did show slight sensitivity to the changes in environmental polarity, as evidenced by an elongation of the fluorescence lifetime when methanol was added to the solution (Table D in S1 File). Similar to the cell-permeable FPT, the control copolymer diffused throughout the cell when incubated at a concentration of 0.01 w/v% in 5 w/v% glucose at 25°C for 20 min. The fluorescence lifetime of the control copolymer in cells did not display any response to increases in the temperature of the medium (Fig. F in S1 File). This result supports the temperature specificity of the cell-permeable FPT and indicates a uniform polarity at the intracellular spaces available to FPTs.

To obtain reproducible measurements of the fluorescence lifetime using time-correlated single-photon-counting FLIM, the collection of a sufficient number of photons at a low photon count rate is necessary [14]. We optimized the scanning condition (as described in Materials and Methods) and set the scanning duration to 60 seconds. During the 60-second scan, the cell-permeable FPT, with DBD as a fluorescent unit [9], loaded into HeLa cells showed significant photobleaching (a 12.6%±5.0% decrease in the photon count rate), while the FPT with DBThD showed only a 6.0%±3.5% decrease in the photon count rate (*n* = 10, respectively).

**Fig 5:**
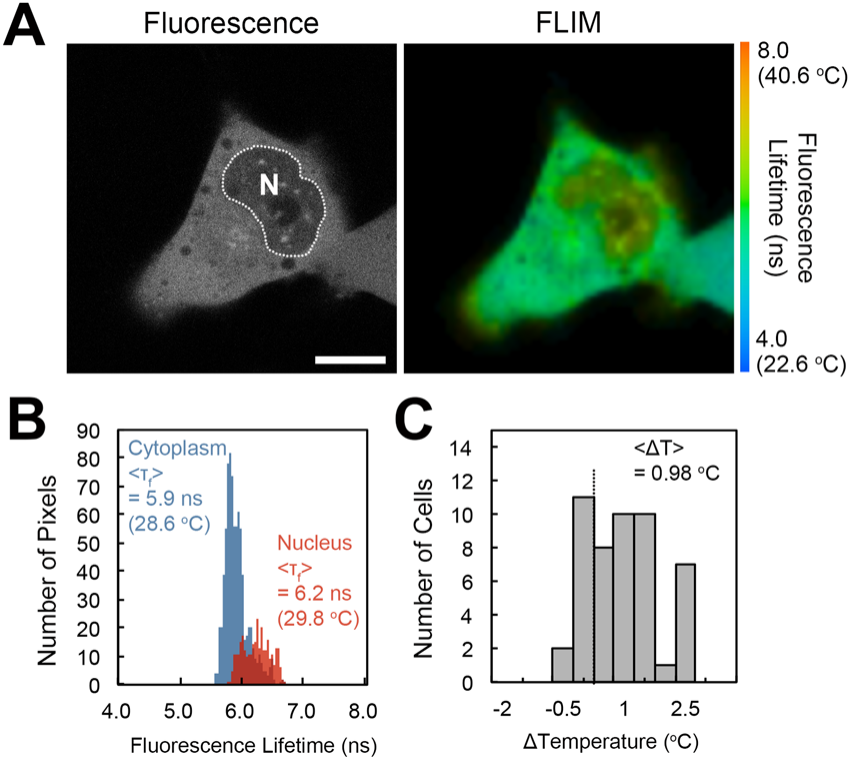
Temperature mapping of living HeLa cells. A) Confocal fluorescence images and fluorescence lifetime images of the cell-permeable FPT in a HeLa cell. N indicates the nucleus (the area of the nucleus is indicated by a dotted line). B) Histograms of the fluorescence lifetime in the nucleus (red) and in the cytoplasm (blue) in a cell in A. C) Histogram of the temperature difference between the nucleus and the cytoplasm (*n* = 49). The temperature difference (<*ΔT*>) was calculated by subtracting the average temperature of the cytoplasm from that of the nucleus. The temperature of the medium was maintained at 30°C. Scale bar = 10 µm.

We then conducted FLIM analysis for intracellular temperature measurement. For intracellular temperature mapping via FLIM, the medium temperature was set at 30°C; the temperature of the specimen was readily maintained at this temperature because of its small difference from the ambient temperature (~24–27°C) [6]. Although the cell-permeable FPT diffused throughout the cell, including the cytosol and the nucleus, many cells displayed lower fluorescence intensity in the nucleus (Fig. 5A, left), suggesting less efficient incorporation of the FPT into the nucleus. Despite this reduced fluorescence intensity, a significant increase in the fluorescence lifetime was evident in the nucleus (Figs. 5A and 5B). The analysis of many cell samples (*n* = 49) revealed that the average temperature difference between the nucleus and the cytoplasm was 0.98°C (Fig. 5C).

**Fig 6:**
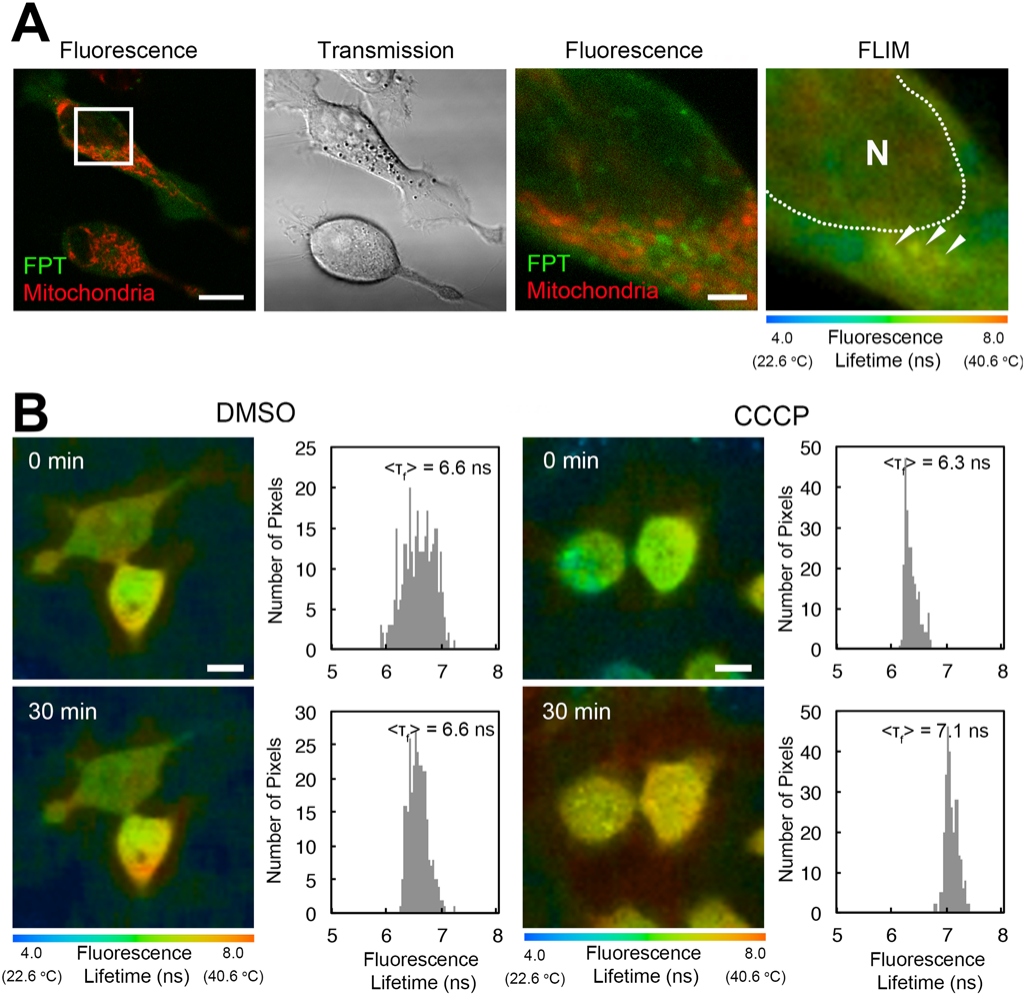
Heat production by the mitochondria in living HeLa cells. A) Confocal fluorescence images of the cell-permeable FPT (green) and MitoTracker Deep Red FM (red), and a transmitted light image, and a FLIM image of HeLa cells. A square in the leftmost image indicates the region of interest, of which fluorescence lifetime was analyzed (in the rightmost figure). Arrowheads in the FLIM image denote local heat production, and N indicates the nucleus (the area of the nucleus is indicated by a dotted line). The temperature of the medium was maintained at 30°C. Scale bars = 10 μm (for the leftmost fluorescence image and transmission image) or 2 µm (for the enlarged fluorescence image at the second from the right and the FLIM image). B) An increase in the intracellular temperature after the inhibition of ATP synthesis by the uncoupler CCCP. FLIM images and histograms of the fluorescence lifetime of cells in the field of view after a treatment of control DMSO (left column) and CCCP (right column). Scale bars = 10 μm.

In our previous study using an FPT that required microinjection for delivery into cells, we detected heat production near the mitochondria [6]. In the present study, we also detected increases in the fluorescence lifetime of the cell-permeable FPT in many cells near the mitochondria (indicated by arrows in Fig. 6A), which were visualized using a mitochondrial indicator. To confirm that the heat production near the mitochondria derives from the mitochondrial function, we treated HeLa cells with an uncoupling reagent CCCP. Uncoupling reagents inhibit ATP synthesis in the mitochondria and induce a release of energy in the form of heat [6,15,16]. The incubation of HeLa cells with 10 µM CCCP significantly increased the cellular temperature, as evidenced by an elongation of the fluorescence lifetime of FPT, while the DMSO control did not induce changes in the fluorescence lifetime (Fig. 6B). The average temperature increase caused by the CCCP treatment was 1.57±1.41°C (average±s.d., 17 CCCP-treated cells from two dishes in one experiment were analyzed. Experiments were repeated twice, and CCCP treatment induced temperature increase in both experiments). In contrast, the control copolymer did not show elongation of the fluorescence lifetime near the mitochondria or in cells after treatment with CCCP (Fig. G in S1 File).

### Application of the cell-permeable FPT to other mammalian cell lines

Finally, we examined whether temperature imaging using this cell-permeable FPT was applicable to other mammalian cell lines. NIH/3T3 (mouse fibroblast-like cell line) and COS7 (African green monkey fibroblast-like kidney cell line expressing SV40 T-antigen) cells were used.

**Fig 7:**
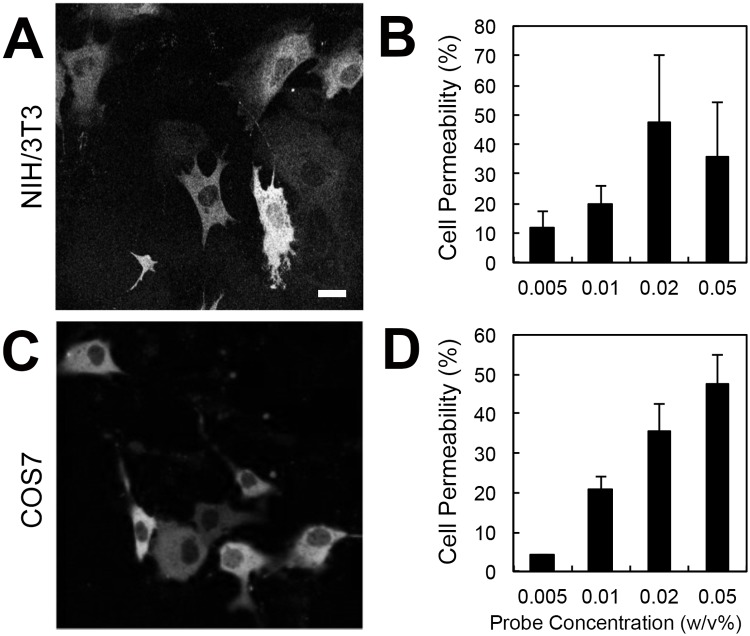
Incorporation efficiency of the cell-permeable FPT into NIH/3T3 and COS7 cells. A, C) Representative confocal fluorescence images of NIH/3T3 cells (A) and COS7 cells (C) treated with 0.02 w/v% FPT. Scale bar = 10 µm. B, D) The concentration-dependent incorporation efficiency of FPT in NIH/3T3 cells (B) and COS7 cells (D).

As shown in Fig. 7, each cell line exhibited a specific incorporation efficiency that depended on the FPT concentration. Treatment with 0.01 w/v% FPT, which was optimal for HeLa cells, resulted in lower incorporation efficiencies into both NIH/3T3 and COS7 cells, and although 0.02 w/v% FPT produced sufficient labeling in NIH/3T3 cells (Figs. 7A and 7B), 0.05 w/v% was more appropriate for COS7 cells (Figs. 7C and 7D). The cell-permeable FPT diffused throughout these cells, although the incorporation efficiency into the nucleus was lower than that into the cytosol in both cell lines (Figs. 7A and 7C). When cells were treated with 0.01 w/v% FPT for 10 min, the average cell viability tested by PI staining showed 94.4±3.7% and 77.8±6.6% for NIH/3T3 and COS7, respectively, in triplicate experiments. The morphology of mitochondria of COS7 cells loaded with FPT was comparable to that of cells without FPT (Fig. H in S1 File), indicating that FPT treatment caused little physical damage to the cells.

**Fig 8:**
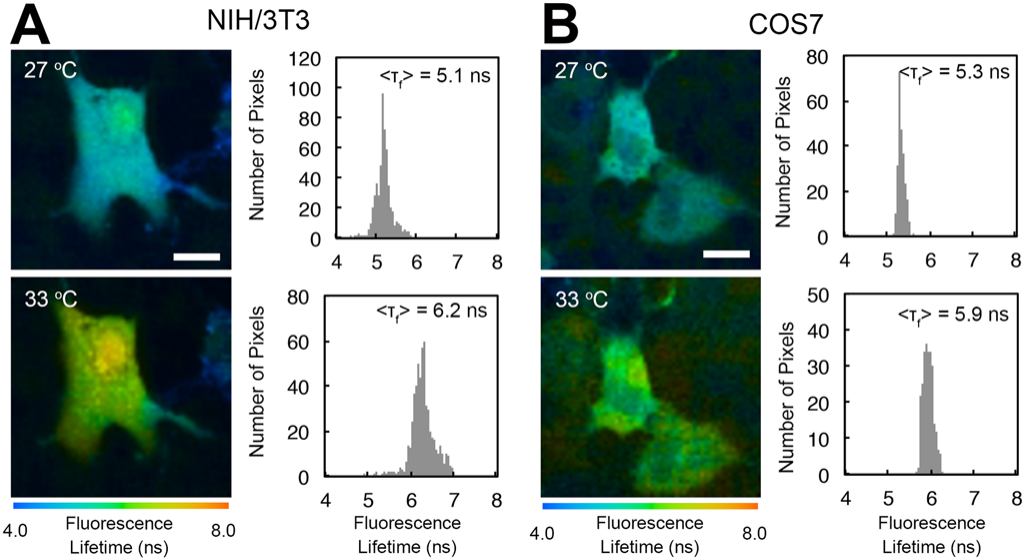
Temperature imaging of living NIH/3T3 and COS7 cells via FLIM using the cell-permeable FPT. A, B) FLIM images and histograms of the fluorescence lifetime of FPT in NIH/3T3 cells (A) and COS7 cells (B). The temperature of the culture medium is indicated in the FLIM images. <τ_f_> indicates the average fluorescence lifetime of cells in the field of view. Scale bars = 10 µm.

FLIM analysis revealed that the fluorescence lifetime of the cell-permeable FPT introduced into NIH/3T3 and COS7 cells elongated as the medium temperature increased (Fig. 8). Although the fluorescence lifetimes of the FPT in NIH/3T3 or COS7 cells were not readily converted to the temperature because the temperature-response of the FPT differs depending on the cell line [6], this result confirmed the functionality of this FPT in these cell types.

## Discussion

In this study, we developed a novel FPT that displays cell permeability and high photostability and established a protocol for delivering this FPT into mammalian cell lines for intracellular temperature mapping. We showed that this FPT, which contains an optimized APTMA level, responded to a temperature range between 25 and 40°C and is therefore suitable for temperature analyses in mammalian cells. Changes in the FPT fluorescence are highly temperature specific, as its response to the temperature is not affected by other environmental changes such as pH, ionic strength, and viscosity. The absence of temperature-dependent changes in the fluorescence lifetime of the control copolymer also supports the temperature specificity of the FPT. FLIM analysis of the cell-permeable FPT in HeLa cells revealed a similar intracellular temperature distribution to that detected previously using a microinjected FPT [6]: the temperature in the nucleus was approximately 1°C higher than that in the cytosol, and the temperature near the mitochondria was higher than that of the rest of the cytoplasm. We also showed that intracellular temperature imaging using this FPT is applicable in many mammalian cell lines. Among the many novel intracellular thermometers reported recently [16–23], our cell-permeable FPT displays an advantage over other methods: its introduction into living cells involves a simple and rapid procedure that does not require special devices. Based on its high temperature specificity and high spatial and temperature resolution, this FPT is expected to contribute to the examination of various biological events in the near future.

Although the exact mechanism by which cell-permeable FPTs are incorporated into living cells is unknown, both endocytosis and chemical permeation could contribute to the introduction of FPTs into cells. We noted that approximately 10% of the cells displayed punctate fluorescence, which could suggest an endocytosis-dependent incorporation pathway. However, the predominant pathway is likely chemical permeation, as this permeation occurred more rapidly than clathrin-dependent endocytosis, as discussed previously [9]. In addition, the treatment of cells at 4°C, a temperature at which various cellular activities, including endocytosis, are hindered, did not inhibit cellular uptake of the FPT. We also confirmed that the percentage of cells displaying punctate fluorescence did not change when the cells were treated at 4°C. Similar endocytosis-independent chemical permeation of macromolecules into cells has been observed with lysine-rich Pep-1 peptide [24], arginine-rich peptide [25], cationic polyfluorenes [26], and carbon nanotubes [27]. Although many of those macromolecules have positively charged surface, it is not clear how this surface character is involved in the cell permeation of those molecules. We were unable to find a correlation between the zeta potential and the incorporation efficiency of FPTs (Table C in S1 File). The size of the FPTs was also not correlated with their incorporation efficiency (Table B in S1 File). In our study, however, we clearly showed a positive correlation between the incorporation efficiency and the ratio of APTMA units in the polymer. In addition, strong inhibition of FPT incorporation into cells by the presence of ions in the solution was shown. Although the mechanism of cellular retention of FPTs is not known, it is possible that the presence of ions at high concentration in a cell inhibits the association between the membrane and the FPTs, thus releasing the FPTs from the cell.

The cytotoxicity of this FPT in mammalian cell lines was closely examined in this study. The absence of cell proliferation in cells treated with this FPT suggests that cell division was likely inhibited. Although the inhibition of cell division has not been previously described [6,9], it was noted that the previously reported FPT inhibited cell division when microinjected into cells before cell division; cell division did proceed, however, when the FPT was microinjected into cells after the initiation of cell division. These results suggest that FPTs are not suitable for investigating cell division or for analyses lasting longer than 1 d. The mechanism by which FPTs inhibit cell division and cause cytotoxicity is unknown, however, it is not likely caused by the specific interaction with FPT and molecules in the cells, such as proteins or DNA, as FPT freely diffuses both in the cytoplasm and in the nucleus (Fig. I in S1 File), and neither fluorescence spectrum (Fig. J in S1 File) nor the fluorescence lifetime (Table E in S1 File) were affected by the presence of DNA. As cells exhibited sufficient viability up to 6 hours after treatment, FPT is applicable for various physiological experiments, such as examining the effect of chemicals, which typically follow a time-course of up to several hours. Due to its ability to be spontaneously incorporated into many cell types within a short treatment time, statistical analyses using many cell samples are possible using this FPT.

Although it is widely accepted that the modification of energy metabolism, including enhanced glycolysis and decreased mitochondrial function, is the hallmark of cancer cells [28,29], more recently it has become apparent that the level of this modification differs depending on the cell line. HeLa cells, for example, were shown to have mitochondrial oxidative metabolism [30,31]. Thus, the local heat generation near the mitochondria observed in this study is likely a result of active energy metabolism by the mitochondria. In the future, it would be of interest to apply intracellular temperature imaging by FPT-FLIM to other cell lines reported to be dependent on glycolysis, such as CT-26 [30], and to compare the intracellular heat maps.

The heat generation in a single cell and the presence of a temperature gradient inside a cell, reported in our current and previous studies, have recently been under debate. Based on theoretical models to estimate heat diffusion and temperature discontinuity in a cell, Baffou et al. argued against the significant generation of heat by a single cell and discussed that the previously reported heat generation by a single cell could be a result of an artifact of cellular thermometers detecting cellular changes in factors other than the temperature [32]. However, the fact that significant heat generation by a single cell upon chemical stimulation has been observed using many intracellular thermometers built based on different principals [6,7,16,19,20,23], as well as using thermometers measuring the temperature of the cellular surface that are thus not affected by the complexity of the interior of a living cell [33–36], is strongly in favor of the idea that significant heat generation by a single cell upon chemical stimulation is indeed possible. Although the temperature difference inside a cell observed by our FPT-FLIM has not been repeated thus far due to a lack of cellular thermometers with high spatial and temperature resolution comparable to FPTs, increasing interest in the temperature measurement of cells [3–5] will enable the introduction of novel cellular thermometers to evaluate the temperature distribution inside of a cell in the near future.

## Supporting Information

S1 File Supporting figures and tables. (DOCX)
