# Supplementary material for: A Cell-Permeable Fluorescent Polymeric Thermometer for Intracellular Temperature Mapping in Mammalian Cell Lines
Source: PLoS One. 2015 Feb 18;10(2):e0117677. doi: 10.1371/journal.pone.0117677 (PMC4333297; doi:10.1371/journal.pone.0117677)
Supplement: S1 File — (DOCX) [file pone.0117677.s001.docx]

**SUPPORTING INFORMATION**

A Cell-Permeable Fluorescent Polymeric Thermometer

for Intracellular Temperature Mapping in Mammalian Cell Lines

Teruyuki Hayashi, Nanaho Fukuda, Seiichi Uchiyama, and Noriko Inada

**Materials and Methods**

**Measurement of temperature-dependent changes in the fluorescence lifetimes of FPT in cell extract by FLIM**

The temperature-dependent change in the fluorescence lifetime of 0.02 w/v% AP4-FPT in a HeLa cell extract was analyzed using TCS-SP5 confocal laser-scanning microscope (Leica) equipped with a 405 laser (PDL 800-B, PicoQuant) and TCSPC module SPC-830 (Becker & Hickl). The pulse repetition rate of 405 laser was set at 20 MHz. The temperature of the microscope stage was regulated using an INUB-F1 controller (Tokai Hit). The temperature of 250 µL sample in a 35-mm glass dish (glass 12 ϕ, Iwaki, Japan) was monitored using a thermocouple probe (TSU-0125 thermometer equipped with a TSU-7225 probe, Tokai Hit). The fluorescence was captured through an HCX PL APO Ibd.BL 63× 1.4 N.A. oil objective (Leica) with zoom factor 1 in 64×64 pixel format at 400 Hz scanning speed (scanning duration was set for 60 seconds) through bandpass 500-700 nm. The obtained fluorescence decay curve was analyzed by fitting the curve by a double exponential function as described in Materials and Methods in the maintext. The calibration curve with AP4-FPT was obtained by approximating the relationship between the averaged fluorescence lifetime of AP4-FPT in a HeLa cell extract (in triplicate) and the temperature to the sixth-degree polynomial (correlation coefficient r=0.996).

$$\tau_{f}\left( T \right)=-2.272\times{10}^{-5}T^{6}+4.02\times{10}^{-3}T^{5}-2.952\times{10}^{-1}T^{4}+11.53T^{3}-2.523\times{10}^{2}+2.937\times{10}^{3}T-1.42\times{10}^{4}$$

where *T* and τ_f_ (*T*) represent the temperature (°C) and the fluorescence lifetime (ns) at *T* °C, respectively. The temperature resolution ($\delta T$) of the FPT was evaluated as described in the maintext.

**Study on the effects of environmental changes on the fluorescence response of FPT**

The effect of pH and ionic strength was analyzed using 150 mM KCl solution, of which pH was changed by the addition of HCl or KOH, and a concentration of KCl in a solution, respectively. The effect of changes in the environmental viscosity on the fluorescence lifetime of the FPT was studied using Ficoll’s solutions with various concentrations. The FPT (0.01 w/v% for study of the effect of viscosity, or 0.02 w/v% for the studies of the effects of pH, ionic strength and DNA), Ficoll (0-40 mg/mL, M.W. 400,000, Sigma), and DNA (0.02 w/v%, fish sperm, Roche) were dissolved in 150 mM KCl solution. The fluorescence lifetime of the solution was measured using a FluoroCube 300U with an ETC-273T temperature controller. The samples were excited with pulsed diode lasers (NanoLED-405L, Horiba, 405 nm or NanoLED-460, Horiba, 456 nm) at a repetition rate of 1 MHz, and emissions longer than 500 nm were collected. The fluorescence spectrum of FPT was measured with FP-6500 spectrofluorometer (JASCO) with or without DNA was recorded with the excitation at 450 nm. The temperature of solution was set at 30 ^o^C.

**Fluorescence Recovery After Photobleaching (FRAP)**

The mobility of the FPT in living cells was investigated by fluorescence recovery after photobleaching (FRAP). Regions of interest either in the cytoplasm or in the nucleus were selected using Olympus FV1000 twin scanner confocal laser scanning system (Olympus, Tokyo, Japan) equipped with UPLS APO 60× NA 1.35 oil objective (Olympus), and bleached using FV5-LDPSU LD405 laser (Olympus) with scanning speed 12.5 µs/pixel for 10 frames. Time-lapse images were acquired with an excitation of 458 Argon laser with a continuous scan over 2.5 minutes for both the cytoplasm and the nucleus. The fluorescence was acquired with zoom factor ranging from 3 to 4 in 1024×512 pixel format at 2 µs/pixel scanning speed through bandpass 475-575 nm. In the image analysis, the ratio of fluorescence intensity of the photobleached spot area to that of unbleached area was calculated for each time point. The time course of fluorescence recovery (*F*(*t*)) was fitted by a single exponential function using Microsoft Excel:

$$F(t)=B_{1}exp({-t}/{\tau_{r}})+B_{2}$$

**
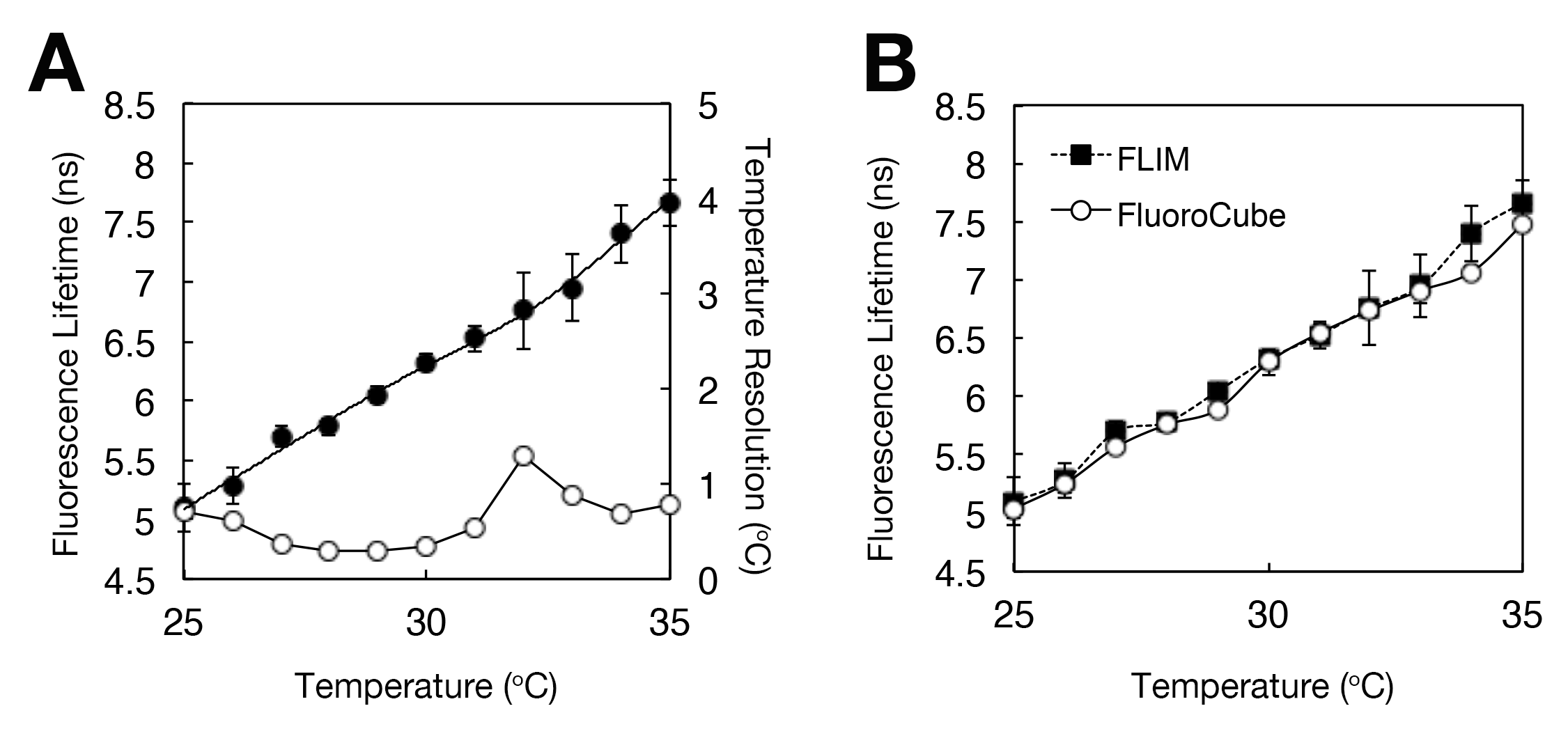
**

**Figure A. Temperature-dependent changes in the fluorescence lifetime measured by FLIM.**

A) Temperature-dependent changes in the fluorescence lifetime of the AP4-FPT (solid circle) and the temperature resolution (open circle) in HeLa cell extracts. The vertical bars indicate the s.d. based on three independent experiments. B) Temperature-dependent changes in the fluorescence lifetime of the AP4-FPT measured by FLIM (solid square, dotted line) and the spectrofluorometer (open circle, solid line).


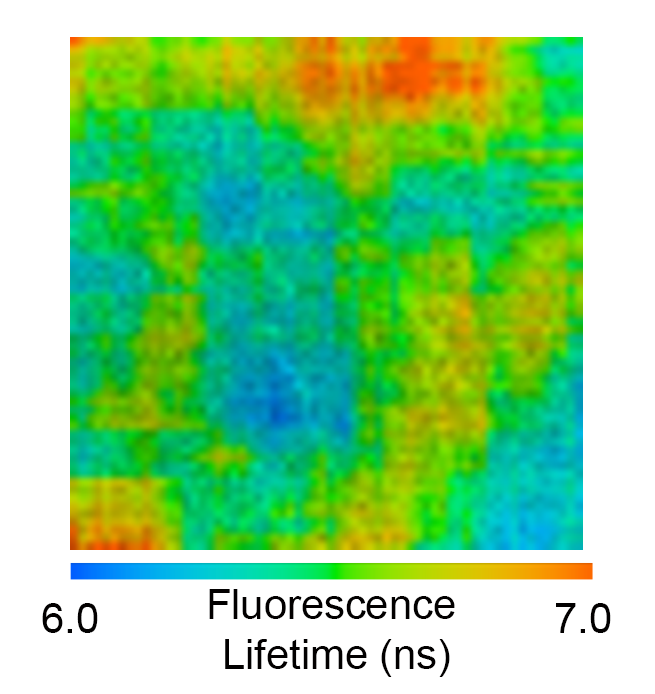


**Figure B. A variation in the fluorescence lifetimes of FPT solution in a microscopic field of view.**

A FLIM image of 0.02 w/v% AP4-FPT in a HeLa cell extract. The temperature of the sample monitored by a thermocouple was set at 30 ^o^C.

**
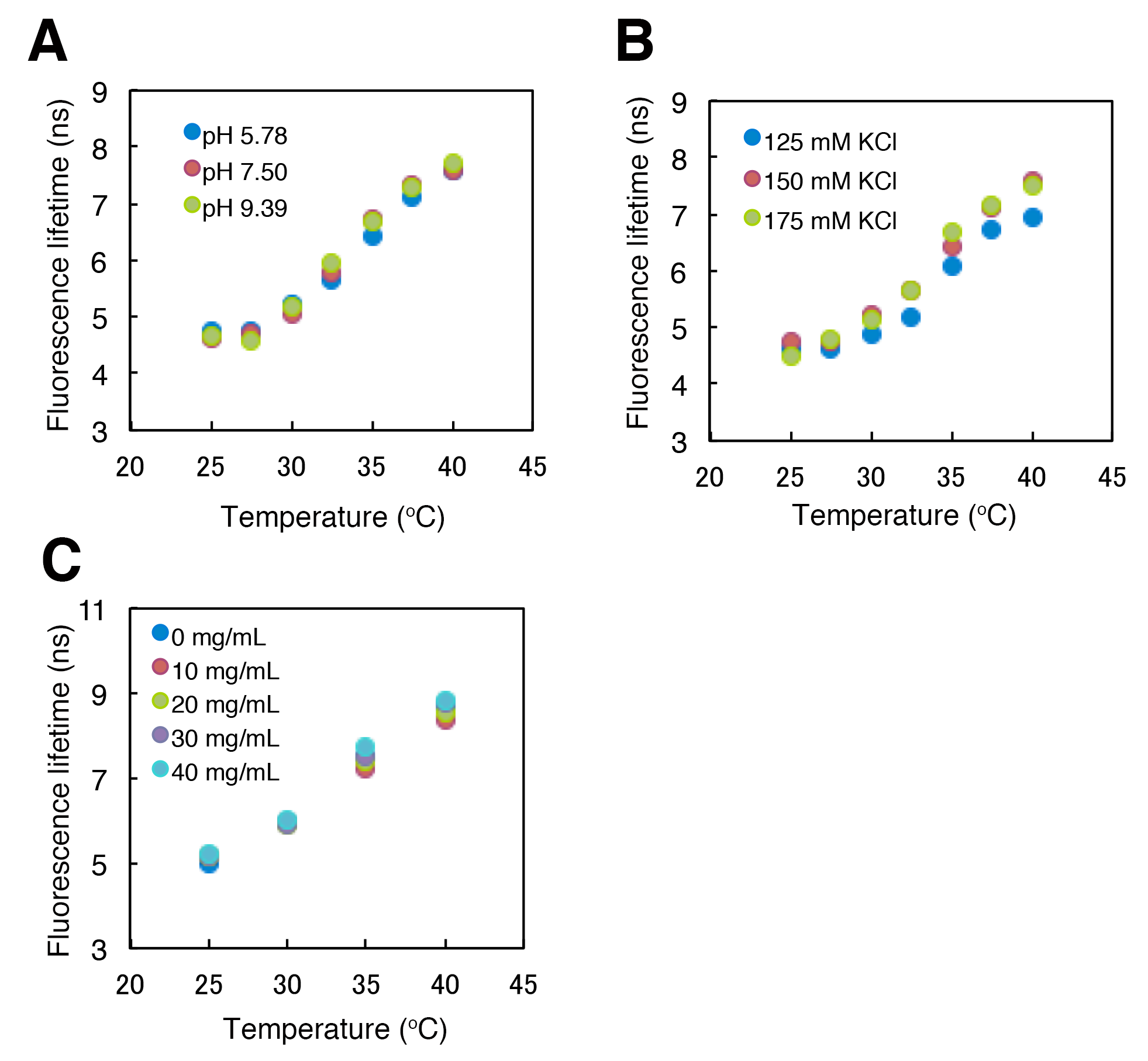
**

**Figure C. Temperature-specificity of the cell-permeable FPT.**

A-C) Effect of environmental pH (A), ionic strength (B) and viscosity (C) on the temperature-dependent response of FPT (0.02 w/v% for A and B, 0.01 w/v% for C). Sample solutions were excited at 405 nm for A and B, and at 456 nm for C.

**
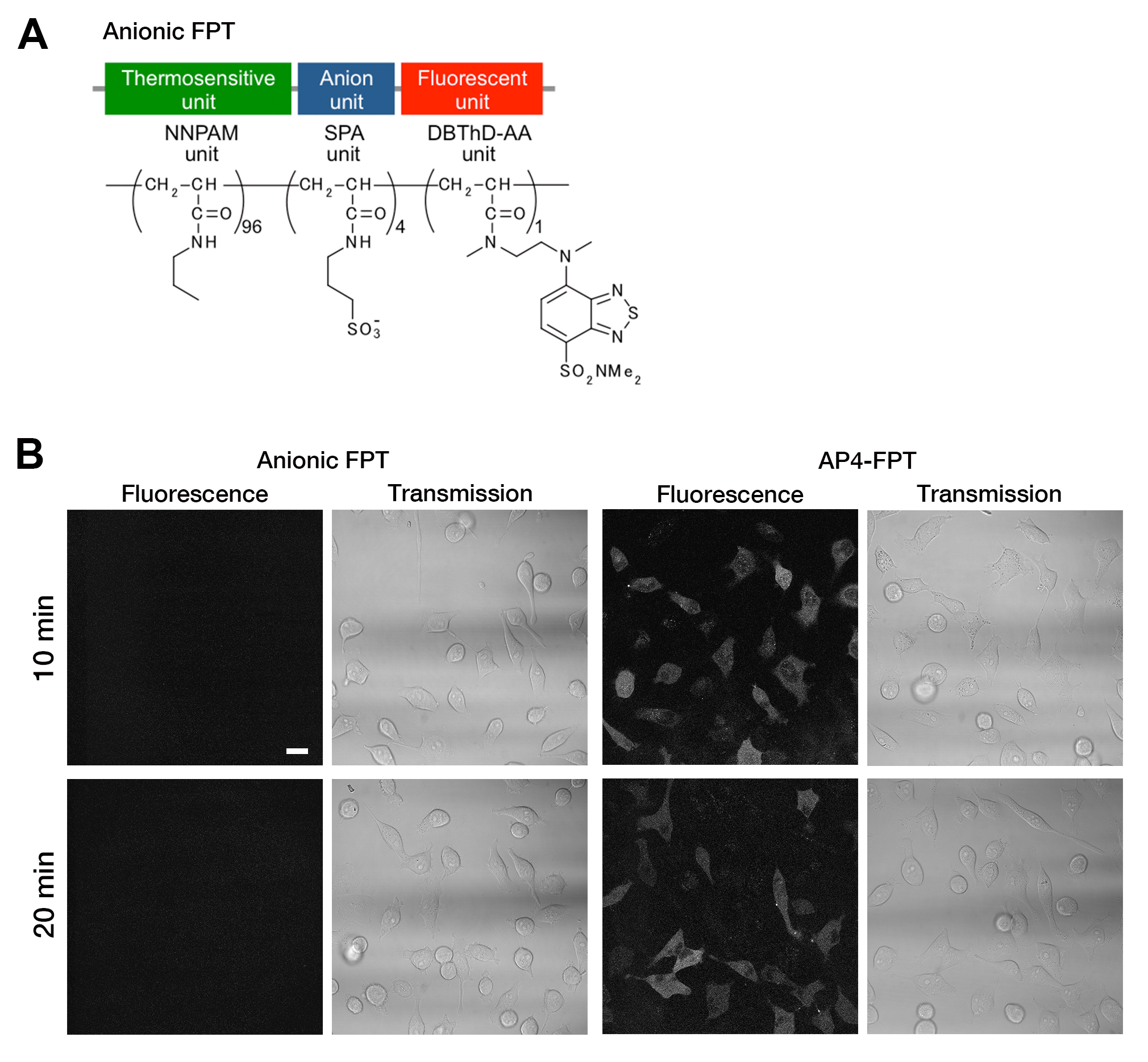
**

**Figure D. Cell-impermeability of the anionic FPT.**

A) Chemical structure of the anionic FPT. An anionic SPA unit was inserted instead of an APTMA unit of the cell-permeable FPT. The original name of each unit is described in the main text. Numbers at each unit indicate the proportion of each unit in the copolymer. B) Confocal fluorescence and transmission microscopy images of HeLa cells treated with 0.01 w/v% of the anionic FPT or 0.01 w/v% of the cell-permeable FPT (AP4-FPT) in 5 % glucose at 25 ^o^C for 10 min or 20 min. Scale bar = 20 µm.

**
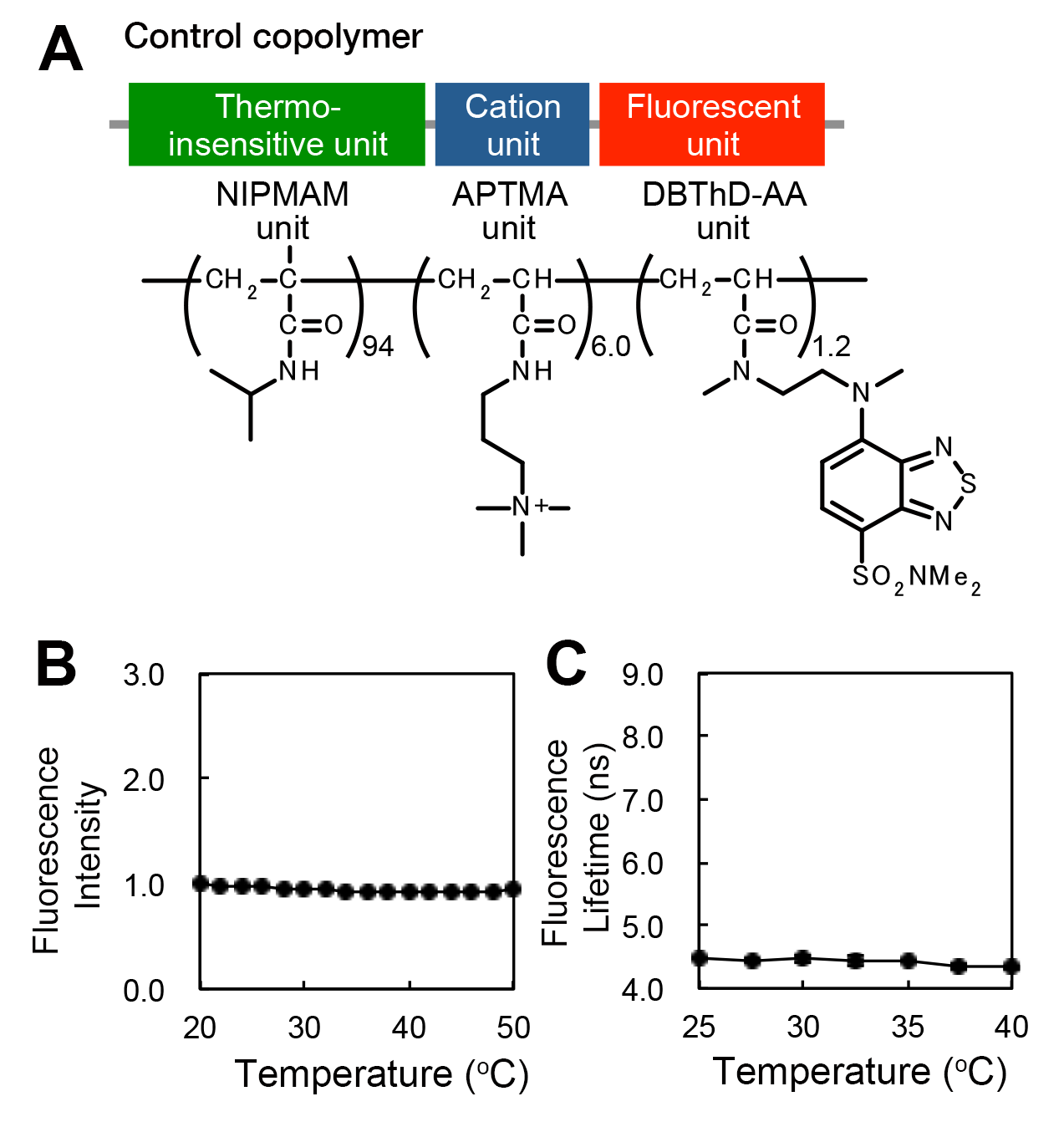
**

**Figure E. Cell-permeable fluorescent control copolymer.**

A) Chemical structure of the cell-permeable fluorescent control copolymer. The original name of each unit is described in the main text. Numbers at each unit indicate the proportion of each unit in the copolymer. B) A temperature-dependent change in the fluorescence intensity of the control copolymer in a HeLa cell extract. C) A temperature-dependent change in the fluorescence lifetime of the control copolymer in a HeLa cell extract. Vertical bars (behind the plots) indicate s.d. from three independent experiments.

**
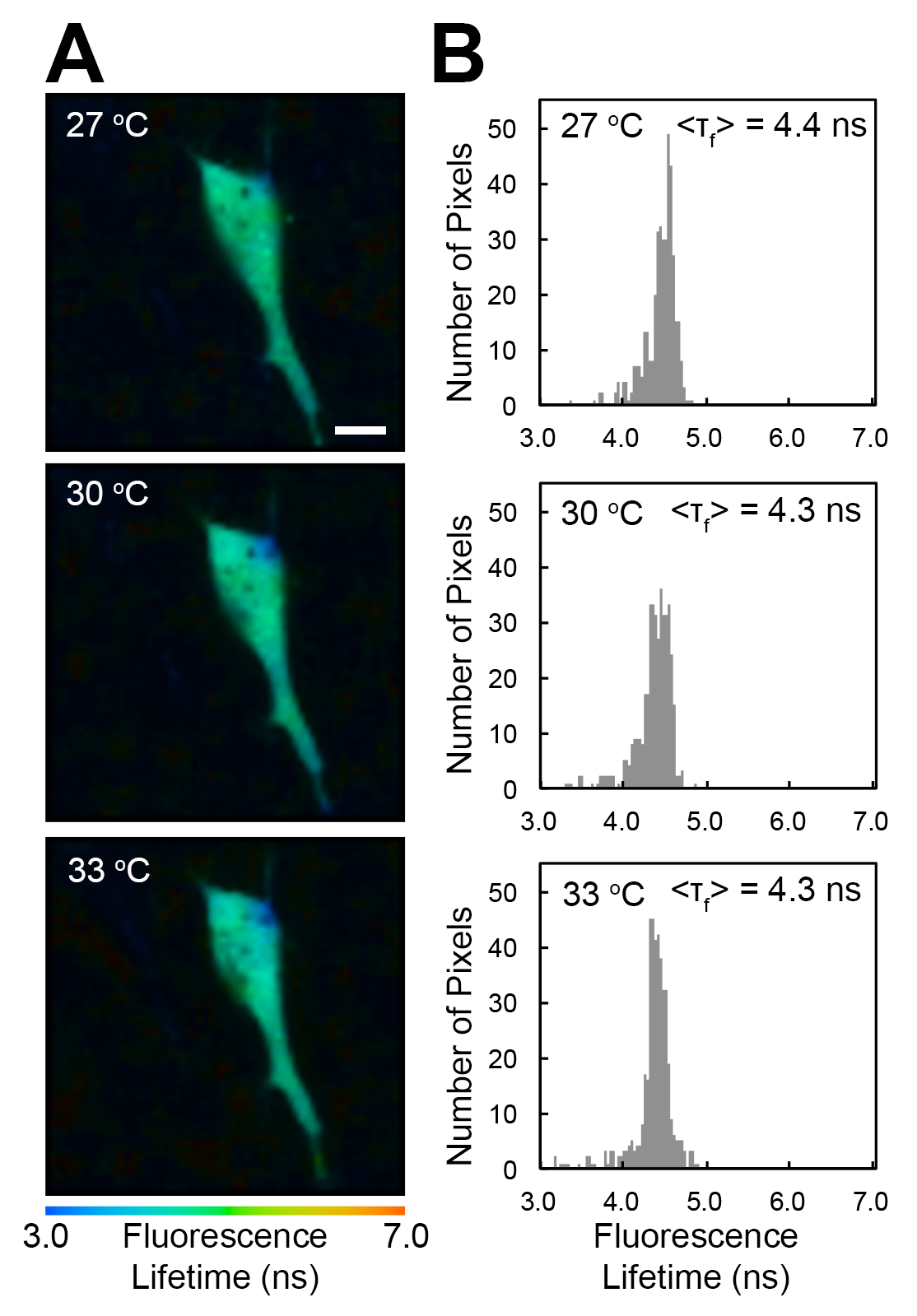
**

**Figure F. Fluorescence lifetime imaging of the control copolymer loaded into living HeLa cells.**

A, B) Florescence lifetime images of the control copolymer (A) and histograms of fluorescence lifetime in a cell (B). The temperature of culture medium was indicated in each image. <τ_f_> indicates an average of the fluorescence lifetime in a cell shown in A. Scale bar = 10 µm.

**
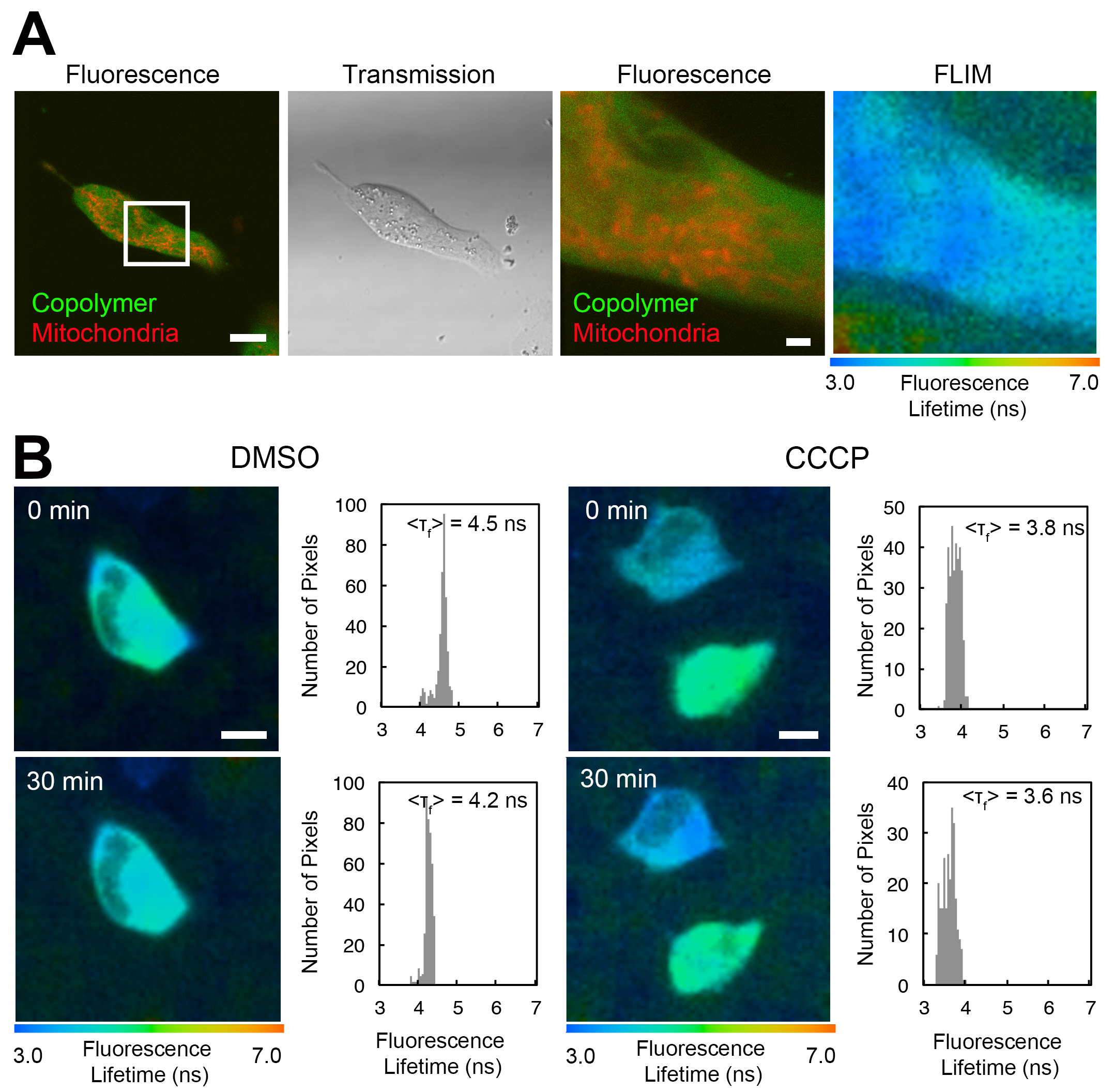
**

**Figure G. Lack of changes in the fluorescence lifetime of the control copolymer near mitochondria or in cells after CCCP treatment.**

A) Confocal fluorescence images of the control copolymer (green) and MitoTracker Deep Red FM (red), and a transmitted light image, and a FLIM image of HeLa cells. A square in the leftmost image indicates the region of interest, of which fluorescence lifetime was analyzed (in the rightmost figure). The temperature of the medium was maintained at 30 °C. Scale bars = 10 μm (for the leftmost fluorescence image and transmission image) or 2 µm (for the enlarged fluorescence image at the second from the right and the FLIM image). B) CCCP and control DMSO treatment on cells loaded with control copolymer. FLIM images and histograms of the fluorescence lifetime of cells in the field of view after a treatment of control DMSO (left column) and CCCP (right column). Scale bars = 10 μm.


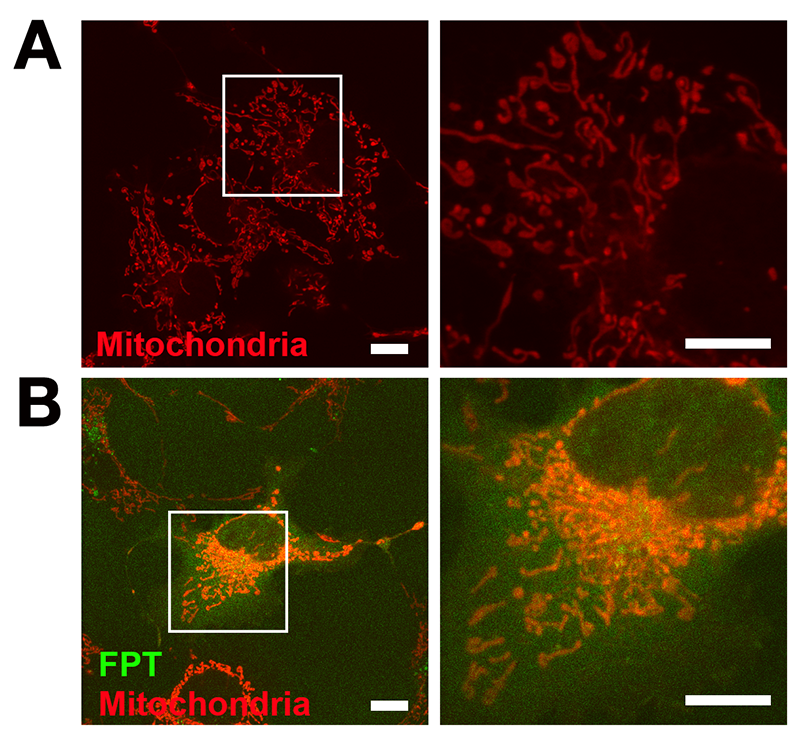


Figure H. The morphology of mitochondria in COS7 cells.

A) Confocal fluorescence images of the mitochondria (stained with MitoTracker Deep Red FM). The region of interest was magnified and shown in the right image. B) Confocal fluorescence images of the FPT (green) and the mitochondria (stained with MitoTracker Deep Red FM, red). The region of interest was magnified and shown in the right image. Scale bars = 10 µm.


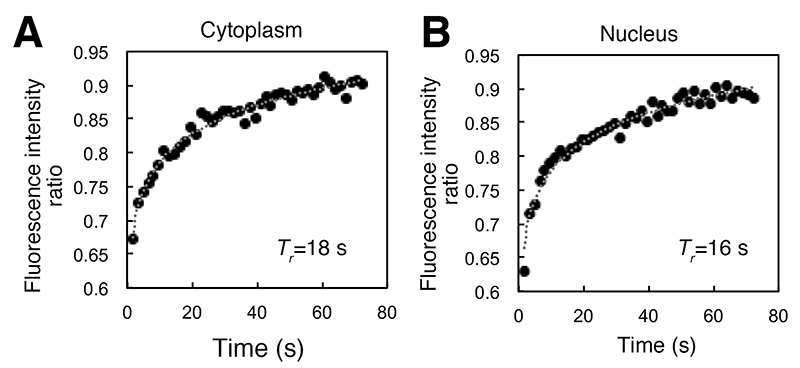


Figure I. Mobility of the cell permeable FPT in HeLa cells.

A, B) The fluorescence recovery of the FPT in the cytoplasm (left) and in the nucleus after photobleaching. Each recovery could be fitted to a single exponential function with the time constant (*T*_r_). Fluorescence intensity ratio represents the ratio of the fluorescence intensity of the photobleached spot area to that of the unbleached cytoplasm or nucleus.


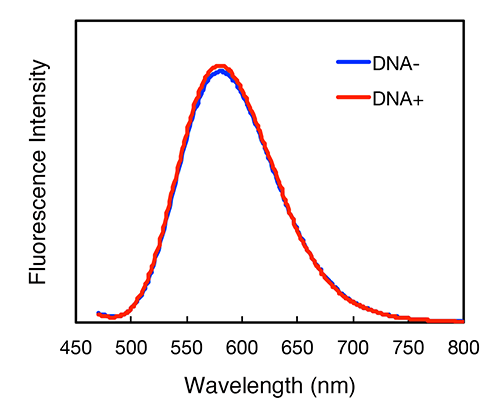


Figure J. The fluorescence spectra of FPT in presence and absence of DNA.

Emission spectra of FPT in 150 mM KCl solution with (red) and without (blue) 0.02 w/v% DNA were obtained with an excitation at 450 nm.

**Table A. Physical properties of the synthesized copolymers.**

| Copolymer | Yield (%) | Composition NNPAM or NIPAM:APTMA or SPA:DBThD) | | Mw^a^ | Mn^b^ |
| --- | --- | --- | --- | --- | --- |
|  |  | In feed | In copolymer |  |  |
| AP2-FPT | 54 | 98:2:1 | 97.3:2.7:0.91 | 23,900 | 10,700 |
| AP4-FPT | 30 | 96:4:1 | 93.8:6.2:0.96 | 12,300 | 6,080 |
| AP8-FPT | 52 | 92:4:1 | 90.0:10.0:0.92 | 5,620 | 3,530 |
| Control copolymer | 38 | 96:4:1 | 94.0:6.0:1.2 | 15,000 | 7,300 |
| Anionic FPT | 50 | 97:3:1 | 96.4:3.6:1.1 | 39,700 | 16,300 |
|  |  |  |  |  |  |
| ^a^Weight-average molecular weight | | |  |  |  |
| ^b^Number-average molecular weight | | |  |  |  |

**Table B. Size of fluorescent polymers in solutions.**

| polymer | 0.01 w/v%, 25°C | 0.01 w/v%, 25°C | 0.01 w/v%, 25°C |
| --- | --- | --- | --- |
|  | in water (nm)^a^ | in PBS (nm)^a^ | in 5% glucose (nm)^a^ |
| AP2-FPT | 5.37 ± 2.46^b^ | 262.42 ± 8.15 | 104.22 ± 7.94 |
| AP4-FPT | 3.42 ± 1.04 | 228.10 ± 9.88 | 118.18 ± 2.68 |
| AP8-FPT | 3.73 ± 1.32 | 173.00 ± 20.51 | ND^c^ |
| Control copolymer | 4.37 ± 1.16 | 7.93 ± 0.76 | ND^c^ |
| Anionic FPT | 4.14 ± 1.13 | 152.74 ± 15.86 | 130.42 ± 8.99 |
|  |  |  |  |
| ^a^Mean ± s.d., *n*=3~10 |  |  |  |
| ^b^15°C, 110.38 ± 1.36 nm at 25°C | |  |  |
| ^c^Could not be determined. Only the signal from glucose was detected. | | | |

**Table C. Zeta potential of fluorescent polymers in solutions.**

| Copolymer | 0.5 w/v% | 0.1 w/v% | 0.1 w/v% |
| --- | --- | --- | --- |
|  | in water (mV)^a^ | in water (mV)^a^ | in 5% glucose (mV)^a^ |
| AP2-FPT | 27.9 ± 0.8^b^ | 20.2 ± 2.6 | 37.6 ± 0.3 |
| AP4-FPT | 30.4 ± 1.4^c^ | 7.5 ± 2.3 | 12.1 ± 3.6 |
| AP8-FPT | 13.7 ± 1.2 | 11.2 ± 2.4 | 19.8 ± 4.7 |
| Control copolymer | 13.6 ± 3.0 |  | 12.0 ± 1.5 |
| Anionic FPT | -42.8 ± 0.8 |  | -36.7 ± 1.9 |
|  |  |  |  |
| ^a^Mean ± s.d., *n*=5~7 at 25 °C |  |  |  |
| ^b^10.2 ± 0.8 nm at 15°C |  |  |  |
| ^c^19.3 ± 0.9 nm at 15°C |  |  |  |

**Table D. The effect of polarity on the temperature response of fluorescent polymers.**

| T(^o^C) | AP4-FPT | | Control copolymer | |
| --- | --- | --- | --- | --- |
|  | in 150 mM KCl (ns) | in 150 mM KCl (water 7: methanol 1, v/v)(ns) | in 150 mM KCl (ns) | in 150 mM KCl (water 7: methanol 1, v/v)(ns) |
| 25 | 4.97 | 6.04 | 5.38 | 5.97 |
| 30 | 5.96 | 6.76 | 5.30 | 5.80 |
| 35 | 7.51 | 7.72 | 5.22 | 5.54 |

**Table E. The effect of DNA on the fluorescence lifetime of the fluorescent polymer.**

|  | 0.02 w/v% DNA+ (ns)^a^ | DNA- (ns)^a^ |
| --- | --- | --- |
| AP4-FPT | 4.97 ± 0.07 | 4.94 ± 0.10 |
|  |  |  |
| ^a^Mean ± s.d., *n*=3 | |  |
